# Multiple Endocrine Neoplasia Type 1 (MEN1) Syndrome Clinical Presentation and the Role of Newer Functional Imaging in the Diagnosis and Management: A Case Report

**DOI:** 10.7759/cureus.79580

**Published:** 2025-02-24

**Authors:** Rishab Singh, Shiv A Goel, Jagadeesh S Singh, Deepa Regina John, Pokhraj P Suthar

**Affiliations:** 1 Department of Radiology, University of Illinois Urbana-Champaign, Champaign, USA; 2 Department of Diagnostic Radiology and Nuclear Medicine, Rush University Medical Center, Chicago, USA; 3 Department of Radiology, Saint Louis University, St. Louis, USA; 4 Department of Radiology, East Suffolk and North Essex NHS Foundation Trust, Colchester, GBR

**Keywords:** ct, ga68, men1, pet/ct, ultrasound (u/s)

## Abstract

Multiple endocrine neoplasia type 1 (MEN1) is a rare genetic disorder characterized by the development of both benign and malignant tumors in the pituitary, parathyroid, and pancreatic glands. These tumors cause hormonal imbalances that lead to various clinical manifestations, including hyperparathyroidism, abdominal pain, and mental disturbances. The management of MEN1 requires accurate detection and localization of these tumors, making imaging a critical tool.

In the management of MEN1, various imaging modalities are employed to detect, monitor, and assess tumor progression. Imaging techniques include conventional imaging such as computed tomography (CT) and magnetic resonance imaging (MRI). However, functional imaging, particularly 68Ga-DOTATATE PET/CT, has emerged as a highly effective approach for detecting neuroendocrine tumors associated with MEN1 and providing crucial data for long-term surveillance.

We present a case of a patient with MEN1 who presented with clinical symptoms of hyperparathyroidism. The patient was found to have adenomas in the pituitary, parathyroid, and pancreatic glands, consistent with the manifestations of MEN1. A combination of imaging techniques, including CT, MRI, and 68Ga-DOTATATE PET/CT scans, was utilized to detect and localize the tumors, guiding further clinical management.

This case highlights the increased importance of functional imaging, particularly 68Ga-DOTATATE PET/CT, in the diagnosis and management of MEN1. The ability to detect and monitor tumors in multiple endocrine glands plays a crucial role in improving patient outcomes through more targeted treatment strategies and long-term surveillance.

## Introduction

Multiple endocrine neoplasia (MEN) is a rare genetic disorder involving genetic mutations that cause the development of benign or malignant tumors in multiple endocrine glands. These tumors cause hormone imbalance leading to various clinical syndromes. MEN type 1 (MEN1) has an estimated prevalence of one in 20,000 to 1 in 40,000. There is no apparent gender bias. MEN1 is autosomal dominant, and more than 95% of individuals with the mutant MEN1 link gene express MEN1 symptoms by age 40-50 [1]. The other MEN types include MEN type 2 and MEN type 4. The clinical overlap between MEN2B and MEN2A, particularly in presenting symptoms such as mucosal neuromas and marfanoid habitus, underscores the challenge of diagnosing MEN2B accurately based solely on clinical presentation [2]. While MEN2A is well-established in the literature, there is a distinct lack of detailed exploration of functional imaging techniques in diagnosing MEN2B, especially when these symptoms are present. Functional imaging, which could play a critical role in early diagnosis and monitoring, has not been fully emphasized in the current research landscape. In contrast, MEN4 is predominantly characterized by the development of endocrine tumors, particularly affecting the parathyroid and/or pituitary glands. However, studies on the role of functional imaging in detecting these tumors are still emerging, and the utility of advanced imaging modalities remains underexplored. Diagnosis of MEN1 involves a combination of clinical assessment, biochemical testing, diagnostic imaging, and genetic analysis. Early diagnosis of MEN is crucial for effective management and improving outcomes. Delayed diagnosis of MEN1 can significantly impact patient outcomes, as tumors may progress to malignant forms before intervention, complicating treatment and worsening prognosis. The lack of widespread awareness and the subtlety of early symptoms contribute to these delays. Genetic counseling for affected individuals and their families to understand risks, implications, and inheritance patterns of these disorders is also important. 

## Case presentation

A 42-year-old woman was referred to a university hospital for management of MEN1 syndrome confirmed genetically. She had previously presented with a history of hyperparathyroidism and nephrolithiasis in an outside hospital. Nephrolithiasis, the presence of kidney stones, is a common symptom of hyperparathyroidism, due to elevated calcium levels in the bloodstream, which deposit in the kidneys. The patient’s blood work lab results then showed a high value for “INTACT PTH” of 367 pg/mL (reference normal range: 15-65 pg/mL), indicating hyperparathyroidism. She underwent parathyroidectomy (with right forearm re-implantation) and right hemithyroidectomy to address the hyperparathyroidism that was caused by MEN1. She was currently being treated for hypoparathyroidism and hypothyroidism post-surgery. She had a family history of MEN1, as her father, sister, and paternal aunt also had confirmed genetic evidence of this disease. 

**Figure 1:**
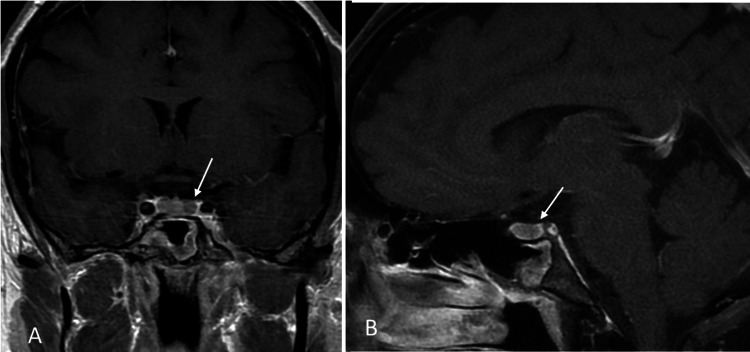
Coronal and sagittal post-contrast MRI (A and B) demonstrate a small hypointense non-enhancing lesion in the pituitary (white arrows) representing a small microadenoma in the patient with MEN1 syndrome

**Figure 2:**
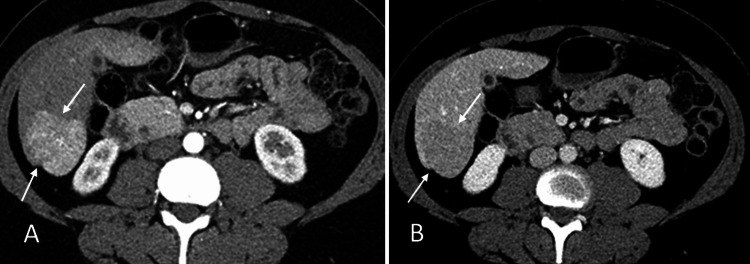
Contrast-enhanced CT abdomen and pelvis. Axial sections (A, B) demonstrate a well-defined enhancing lesion in the inferior right lobe of the liver with a central scar and washout of contrast on the delayed scan (white arrows in A and B) suggestive of focal nodular hyperplasia (FNH).

**Figure 3:**
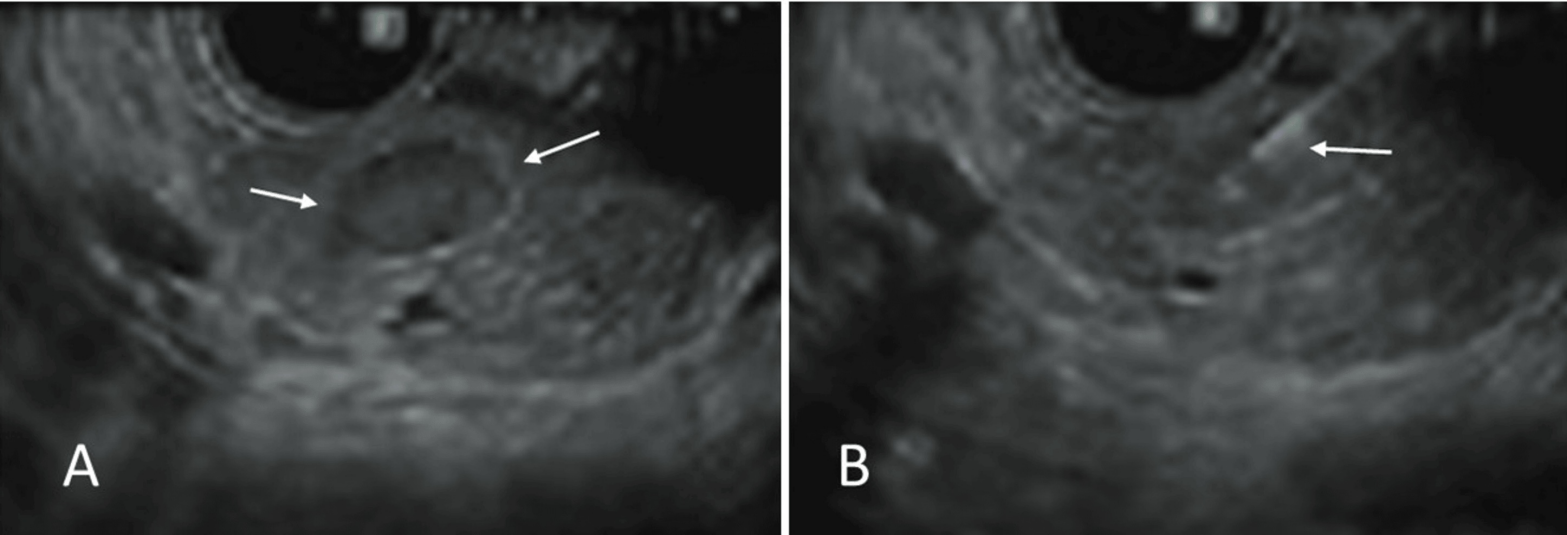
Endoscopic ultrasound demonstrates a well-defined hypoechoic lesion in the pancreas (white arrows in A), which was biopsied (white arrow in B). The histopathology confirmed the neuroendocrine tumor of the pancreas.

**Figure 4:**
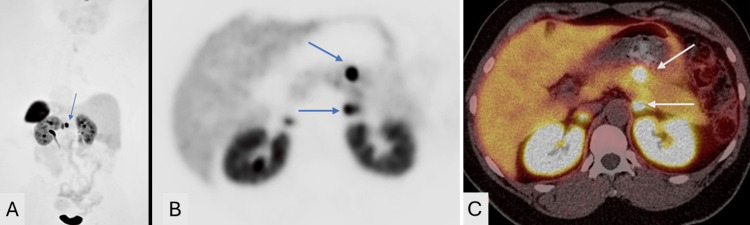
MIP coronal PET image (A), axial attenuation corrected (B), and axial fused images (C) show somatostatin avid lesions in the body of the pancreas (blue and white arrows) representing pancreatic neuroendocrine tumors in the patient with MEN-1 syndrome. MIP: Maximum intensity projection

As part of the screening for MEN 1 syndrome, the patient underwent MRI imaging of the brain and pituitary, and she was found to have a pituitary microadenoma (Figure 1). Other lab findings included elevated pancreatic polypeptide levels at 1959.0 pg/mL (reference normal range 56-480 pg/mL). Gastrin and glucagon were within the normal range at 73 pg/mL and 53, respectively, (reference normal range of gastrin: <100 pg/mL, and reference normal range of glucagon: 50-100 pg/mL). Somatomedin-C was 158 ng/mL (reference normal range of 94 to 267 ng/mL) and the IGF-1 Z score was 0.3 (the normal Z-score is between -2.0 and +2.0). In view of her elevated pancreatic polypeptide levels, she underwent a CT scan of the abdomen which showed an enhancing lesion in the liver with features suggestive of focal nodular hyperplasia (FNH) (Figure 2) and a small suspicious enhancing lesion in the pancreas. She then underwent endoscopic ultrasound which showed two rounded lesions in the pancreas which were biopsied (Figure 3), and histopathology showed neuroendocrine tumors (NETs). She then underwent PET imaging with 68Ga-DOTATATE PET/CT scans for staging which showed four somatostatin avid lesions in the pancreas confirming the presence of NET foci in the pancreas (Figure 4). There was no significant tracer uptake in the liver lesion seen on the CT scan to suggest metastasis. There was no other local or distant metastasis on the PET scan. The patient is currently being referred to Surgical Oncology for consideration of surgical management of the pancreatic NETs. We obtained the institutional waiver for case report submission (Form 118) from the Institutional Review Board (IRB) on 10/24/2024. A summary of clinical findings and imaging results is displayed in Table 1.

**Table 1:** Summary of clinical findings and imaging results MEN1: Multiple endocrine neoplasia type 1

Clinical Finding	Result/Observation
Patient age	42 years old
Diagnosis	MEN1 syndrome (genetically confirmed)
Presenting symptoms	Hyperparathyroidism, nephrolithiasis
Family history	Father, sister, and paternal aunt with confirmed MEN1
Lab findings	Elevated “INTACT PTH”: 367 pg/mL; elevated pancreatic polypeptide: 1959.0 pg/mL; gastrin: 73 pg/mL; glucagon: 53 pg/mL; somatomedin-C: 158 ng/mL; IGF-1 Z score: 0.3
Surgical procedures	Parathyroidectomy (with right forearm re-implantation), right hemithyroidectomy
Post-surgical management	Treatment for hypoparathyroidism and hypothyroidism
Imaging Results	
MRI (brain and pituitary)	Pituitary microadenoma (Figure 1)
CT scan (abdomen)	Enhancing lesion in the liver suggestive of focal nodular hyperplasia (Figure 2); small suspicious enhancing lesion in the pancreas
Endoscopic ultrasound	Two rounded lesions in the pancreas, biopsied (Figure 3), histopathology: neuroendocrine tumor
PET/CT (68Ga-DOTATATE)	Four somatostatin avid lesions in the pancreas (Figure 4), no significant tracer uptake in liver lesion, no metastasis detected
Current referral	Surgical oncology for consideration of pancreatic neuroendocrine tumor resection

## Discussion

MEN1 is an autosomal dominant condition caused by inactivating mutations of the tumor suppressor gene MEN1, resulting in an abnormal protein called menin [3]. This causes benign and malignant tumors of the parathyroid glands, the pituitary gland, and the pancreatic islets. This is also known as Wermer’s syndrome. When involving the parathyroid glands, it can cause primary hyperparathyroidism from hyperplasia or adenoma. This is the most common and usually the earliest manifestation of MEN1, occurring in about 90% of patients. Symptoms can include hypercalcemia (high blood calcium levels), kidney stones, bone pain, osteoporosis, fatigue, muscle weakness, abdominal pain, and mental disturbances [4]. 

If MEN1 involves the pancreatic islets, it can cause gastroenteropancreatic neuroendocrine tumors (GEP-NETs) such as insulinoma, gastrinoma, glucagonoma, VIPoma, or somatostatinoma. Insulinomas produce excess insulin, leading to hypoglycemia. Symptoms include sweating, tremors, palpitations, confusion, or even seizures due to low blood sugar. Gastrinomas produce Zollinger-Ellison syndrome from excess gastrin which can lead to peptic ulcers. Symptoms include abdominal pain, diarrhea, and gastroesophageal reflux. Glucagonomas produce excess glucagon with symptoms including diabetes, weight loss, and necrolytic migratory erythema (skin rash). VIPomas produce vasoactive intestinal peptides and cause severe watery diarrhea, hypokalemia, and dehydration (Verner-Morrison syndrome). Somatostatinomas produce excess somatostatin causing symptoms of diabetes, gallstones, diarrhea, and steatorrhea (fatty stools) [1].

When MEN1 involves the pituitary gland, pituitary adenomas develop. Prolactinomas produce excess prolactin with symptoms of galactorrhea (milk discharge from breasts), amenorrhea (absence of menstrual periods), infertility, and sexual dysfunction (Singh). Growth hormone-secreting tumors can cause acromegaly in adults or gigantism in children. Symptoms include enlarged hands and feet, joint pain, coarse facial features, and cardiovascular issues. Adrenocorticotropic hormone (ACTH)-secreting adenomas produce excess ACTH, leading to Cushing's disease manifesting as weight gain, high blood pressure, diabetes, and osteoporosis [4]. 

Other manifestations of MEN1 include adrenal adenomas, carcinoid tumors, or dermatological manifestations. Outside of endocrine manifestations, common tumors include meningiomas, ependymomas, lipomas, angiofibromas, collagenomas, and leiomyomas. Carcinoid tumors typically occur in the thymus, lungs, or gastrointestinal tract with symptoms including cough, chest pain, and carcinoid syndrome (flushing, diarrhea, wheezing). Dermatological manifestations include angiofibromas, collagenomas, and lipomas mainly cosmetic concerns [3]. 

Diagnosing MEN1 involves a combination of clinical assessment, biochemical testing, diagnostic imaging, and genetic analysis. Early diagnosis of MEN is crucial for effective management and improving outcomes. Genetic counseling for affected individuals and their families to understand risks, implications, and inheritance patterns of these disorders is also important [4]. 

Diagnostic imaging is critical in the evaluation of possible tumors. Neck ultrasound or TC-99m Sestamibi scan evaluates parathyroid glands for hyperplasia or adenomas [5]. MRI or CT scans detect and characterize pituitary adenomas and pancreatic neuroendocrine tumors (pNETs). Endoscopic ultrasound identifies small pNETs. An octreotide scan (somatostatin receptor scintigraphy) and currently DOTATATE PET scan are used to locate NETs expressing somatostatin receptors including in the pancreas, gastrointestinal tract, and thymus [5]. 

The diagnostic criteria are the presence of two or more primary MEN1-related endocrine tumors such as parathyroid, pancreatic, or pituitary tumors [4]. Familial diagnosis is the presence of one MEN1-related endocrine tumor in an individual with a first-degree relative with confirmed MEN1 [1].

Diagnostic imaging plays an important role in the management of MEN1 patients to identify the tumors and affects early surgical or medical management. 

Parathyroid adenomas

Nuclear medicine scintigraphy with Tc -99m Sestamibi scan can help detect parathyroid adenomas. 4D CT of the neck is very helpful in detecting small adenomas or hyperplasia in scintigraphy negative scans. 

Pituitary adenomas

MRI provides a greater distinction between lesions compared to CT and other modalities. The characterization of pituitary adenomas as microadenomas and macroadenomas plays a key role in the implementation of MRI imaging. Microadenomas are more common and are functional in the secretion of hormones, leading to specific symptoms in correlation with the specific hormone produced by the tumor.

Pancreatic neuroendocrine tumors

Cross-sectional imaging methods such as CT or MRI sometimes have difficulties in detecting pNETs depending on the size and blood flow. Newer functional imaging modalities such as Somatostatin receptor imaging with Octreoscan and 68GA-DOTATATE PET/CT are the current mainstay in diagnosing and detecting pNETs [5]. 

Somatostatin receptor positron emission tomography/computed tomography (SSTR PET/CT) has had significant success in detecting pNETs. For instance, studies have demonstrated the benefits of the higher sensitivity of the SSTR PET/CT, allowing for clearer localization of pNETs like insulinomas expressing SSTR2 receptors [6,7]. 

In this case, the liver lesion observed on CT imaging exhibited an enhancing lesion with an arterial phase appearance suggestive of a NET. However, in the delayed phase, the lesion demonstrated a central scar, which is characteristic of FNH, a benign lesion. The central scar seen on delayed imaging, coupled with the overall imaging appearance, strongly pointed toward FNH, which is a common benign liver condition that can present similarly to more concerning lesions in the arterial phase. Because of this imaging pattern, the lesion did not demonstrate significant tracer uptake on PET/CT scanning, as FNH typically does not exhibit the somatostatin avidity that is commonly seen in NETs. This lack of tracer uptake on PET/CT further reinforced the benign nature of the lesion. In contrast, the pancreatic lesions were highly suspicious for NETs, which are closely associated with MEN1 syndrome. These lesions showed somatostatin avidity on PET/CT imaging, a key characteristic of NETs, as they typically express somatostatin receptors. The tracer uptake seen in the pancreatic lesions confirmed their malignant potential, making them a focus for further investigation and surgical planning.

Treatments for patients with MEN1-associated conditions mostly include surgery in combination with appropriate therapeutic medications. pNETs are the leading cause of death among patients with MEN1, which have a higher potential of becoming malignant and initiating metastatic spread throughout the body. Again, surgery is the most likely curative treatment, to avoid metastasis and thereby require pancreatectomy [8]. Somatostatin receptor PET/computed tomography (SSTR PET/CT) plays an important role in initial detection, staging, assessing treatment response, and long-term surveillance of these tumors. 

The prognosis for patients with MEN1 conditions depends highly on the location and severity of the tumors. Patients with nonfunctioning pNETs are given a worse prognosis than functioning tumors such as insulinomas due to their initial asymptomatic nature and higher prevalence at later ages, delaying possible recognition of a lesion and necessary medical intervention [9-11]. The increased size of the tumors also results in a greater likelihood of the development of metastatic disease, which is also related to poor prognosis of patients diagnosed with MEN1-like symptoms. 

## Conclusions

The case highlights the complex clinical presentation of MEN1, underscoring the pivotal role of newer imaging techniques in its diagnosis and management. MEN1 involves a spectrum of endocrine tumors that necessitate early and precise detection to optimize patient outcomes. Advanced imaging modalities, particularly functional imaging such as 68Ga-DOTATATE PET/CT, have revolutionized the evaluation of neuroendocrine tumors, offering higher sensitivity and specificity compared to conventional imaging techniques. These modalities enable early localization, staging, and long-term surveillance of MEN1-associated tumors, thereby guiding targeted treatment strategies. The multidisciplinary management of MEN1, involving endocrinologists, radiologists, surgeons, and genetic counselors, ensures comprehensive care. Genetic counseling remains integral, given the hereditary nature of MEN1, to inform affected families about the risks and preventive strategies. This case further reinforces the importance of a tailored imaging approach, combining anatomical and functional modalities, for identifying clinically significant lesions. The study's limitations, including its single-case design and the lack of long-term outcome data, are not fully addressed. Additionally, the suggestions for future research remain vague; more specific proposals, such as comparing imaging modalities across larger cohorts, would help to strengthen the conclusions.
